# Multiple therapeutic peptide vaccines consisting of combined novel cancer testis antigens and anti-angiogenic peptides for patients with non-small cell lung cancer

**DOI:** 10.1186/1479-5876-11-97

**Published:** 2013-04-11

**Authors:** Hiroyuki Suzuki, Mitsuro Fukuhara, Takumi Yamaura, Satoshi Mutoh, Naoyuki Okabe, Hiroshi Yaginuma, Takeo Hasegawa, Atsushi Yonechi, Jun Osugi, Mika Hoshino, Takashi Kimura, Mitsunori Higuchi, Yutaka Shio, Kazuya Ise, Kazuyoshi Takeda, Mitsukazu Gotoh

**Affiliations:** 1 Department of Regenerative Surgery, Fukushima Medical University, School of Medicine, 1 Hikarigaoka, Fukushima 960-1295, Japan; 2 Department of Immunology, Juntendo University, School of Medicine, 2-1-1 Hongo Bunkyo-ku, Tokyo 113-8421, Japan

**Keywords:** Cancer vaccine, Multiple peptides, Prognosis, Non-small cell lung cancer

## Abstract

**Background:**

Vaccine treatment using multiple peptides derived from multiple proteins is considered to be a promising option for cancer immune therapy, but scientific evidence supporting the therapeutic efficacy of multiple peptides is limited.

**Methods:**

We conducted phase I trials using a mixture of multiple therapeutic peptide vaccines to evaluate their safety, immunogenicity and clinical response in patients with advanced/recurrent NSCLC. We administered two different combinations of four HLA-A24-restricted peptides. Two were peptides derived from vascular endothelial growth factor receptor 1 (VEGFR1) and 2 (VEGFR2), and the third was a peptide derived from up-regulated lung cancer 10 (URLC10, which is also called lymphocyte antigen 6 complex locus K [LY6K]). The fourth peptide used was derived from TTK protein kinase (TTK) or cell division associated 1 (CDCA1). Vaccines were administered weekly by subcutaneous injection into the axillary region of patients with montanide ISA-51 incomplete Freund’s adjuvant, until the disease was judged to have progressed or patients requested to be withdrawn from the trial. Immunological responses were primarily evaluated using an IFN-gamma ELiSPOT assay.

**Results:**

Vaccinations were well tolerated with no severe treatment-associated adverse events except for the reactions that occurred at the injection sites. Peptide-specific T cell responses against at least one peptide were observed in 13 of the 15 patients enrolled. Although no patient exhibited complete or partial responses, seven patients (47%) had stable disease for at least 2 months. The median overall survival time was 398 days, and the 1- and 2-year survival rates were 58.3% and 32.8%, respectively.

**Conclusion:**

Peptide vaccine therapy using a mixture of four novel peptides was found to be safe, and is expected to induce strong specific T cell responses.

**Trial registration:**

These studies were registered with ClinicalTrials.gov NCT00633724 and NCT00874588.

## Background

Lung cancer is the leading cause of cancer death in the world [1]. Despite the recent development of novel treatment modalities for patients with non-small cell lung cancer (NSCLC), survival rates are still unsatisfactory [2]. Furthermore, although molecular-targeted drugs are expected to cause fewer serious adverse events associated with the use of cytotoxic chemotherapeutic agents, but still cause some [3,4]. Therefore, the development of more effective and less toxic therapeutic modalities is eagerly awaited. In this regard, cancer immunotherapy is considered to be a promising option with minimum toxicity, but its effectiveness has not yet been proven to be superior to the presently available treatments. However, several ongoing clinical trials that are administering vaccines, such as MAGE-A3 or BLP25 for lung cancer as an adjuvant treatment or in a maintenance setting after standard chemotherapy, seem to be very promising [5,6]. Although these lung cancer trials have involved the administration of a single vaccine, a combination of multiple peptide vaccines has also been used in several types of solid cancer [7,8].

We have previously identified novel cancer-testis antigens, including up-regulated lung cancer 10 (URLC10; also called lymphocyte antigen 6 complex locus K [LY6K]) [9], TTK protein kinase (TTK) [10] and the cell division cycle associated gene 1 (CDCA1) [11], that were found to be expressed at very high levels in lung cancer using the genome-wide cDNA microarray method. We have also previously reported peptide vaccines that target VEGFR1 [12] and VEGFR2 [13]. To induce a higher level of cytotoxic T lymphocytes (CTLs), also known as cytotoxic T cells, that have direct cancer cell killing activity or block the blood supply to cancer cells, we attempted to combine the peptides derived from cancer-testis antigens, as well as those designed to induce an anti-angiogenic effect to achieve an effective response in patients with advanced NSCLC. In the current study we report on the safety of combination therapy involving multiple peptides and a possible improvement in patient prognosis.

## Methods

### Study design

We performed two phase I clinical trials using two different combinations of peptide vaccines. In the first trial, we administered peptides derived from URLC10, TTK, VEGFR1 and VEGFR2, and in the second trial we administered peptides derived from URLC10, CDCA1, VEGFR1 and VEGFR2. All peptides were restricted to HLA-A*2402. Fifteen HLA-24-positive patients with NSCLC who failed to respond to the standard therapy were enrolled in the three patient/dose/cohort phase I trial involving 0.5, 1 or 3 mg/body for each peptide (for trial 1), or 1 or 3 mg/body for each peptide (for trial 2). The clinical characteristics and treatment information for all patients enrolled in the study are summarized in Table 1. Vaccines were administered weekly and the sites of vaccination were rotated weekly. Administration was by subcutaneous injection into the patient’s axillary region after mixing with incomplete Freund’s adjuvant (IFA) Montanide ISA 51, SEPPIC until progression of the disease was observed, or until the patient declined the continuation of the vaccine treatment. Immunological responses were evaluated by means of INF-gamma ELISPOT assays. Every measurable lesion was evaluated using response evaluation criteria in solid tumors (RECIST) 1.0, and the toxicities caused by the vaccination therapy were assessed using Common Terminology Criteria for Adverse Events (CTCAE) version 3. These studies were approved by the ethical committee of Fukushima Medical University (trial 1 approval number: 554; trial 2 approval number: 810) and were registered with ClinicalTrials.gov (trial 1: NCT00633724; trial 2: NCT00874588). Written informed consent was obtained from all individuals. The trials were carried out in accordance with the Helsinki declaration on experimentation on human subjects.

**Table 1:** Patient clinical characteristics

**Patients**	**Age/Gender (M/F)**	**Stage**	Histology*	**Lesion§**	**Performance status (ECOG)**	**Peptides†**	**Dose (mg)**	Phase of treatment (Prior therapy**)
1	54/M	Recurrence	AD	LN, bone	2	L, T, R1, R2	0.5	5^th^ (PLT, RT)
2	48/M	IIIB	AD	PM, effusion	2	L, T, R1, R2	0.5	5^th^ (PLT)
3	65/M	Recurrence	AD	PM	2	L, T, R1, R2	0.5	6^th^ (PLT, EGFR-TKI)
4	58/M	IV	AD	Primary, bone	2	L, T, R1, R2	1	4^th^ (PLT)
5	60/M	IV	AD	Primary, LN	1	L, T, R1, R2	1	3^rd^ (PLT)
6	47/M	IV	AD	Primary, LN, ADR	0	L, T, R1, R2	1	3^rd^(PLT, RT)
7	40/M	IIIA	AD	Primary, LN	1	L, T, R1, R2	3	3^rd^(PLT)
8	69/M	Recurrence	SQ	PM	1	L, T, R1, R2	3	3^rd^(PLT, RT)
9	65/M	Recurrence	AD	Dissemination	0	L, T, R1, R2	3	2^nd^(PLT, RT)
10	57/M	Recurrence	PLEO	LN	1	L, C, R1, R2	1	3^rd^(PLT, RT)
11	55/F	IIIB	AD	Primary, LN, effusion	2	L, C, R1, R2	1	5^th^(PLT, EGFR-TKI)
12	62/M	Recurrence	AD	PM	1	L, C, R1, R2	1	2^nd^(PLT)
13	68/F	IV	AD	Primary, bone, effusion	2	L, C, R1, R2	3	2^nd^(PLT)
14	39/F	IV	NSCLC	Primary, liver, bone	2	L, C, R1, R2	3	2^nd^(PLT, RT)
15	61/M	Recurrence	AD	PM, LN	1	L, C, R1, R2	3	5^th^(PLT, RT, EGFR-TKI)

*AD: adenocarcinoma; SQ: squamous cell carcinoma; PLEO: pleomorphic carcinoma; NSCLC: non-small cell lung cancer in which further histological determination was not possible.

§LN: lymph nodes metastasis; bone: bone metastasis; PM: pulmonary metastasis; effusion: malignant pleural effusion; Primary: primary tumor; ADR: adrenal gland metastasis; Dissemination: pleural dissemination; liver: liver metastasis.

† L: LY6K; T: TTK; R1: VEGFR1; R2: VEGFR2; C: CDCA1.

**PLT: platinum containing chemotherapy; RT: radiotherapy; EGFR-TKI: epidermal growth factor receptor tyrosine kinase inhibitor.

### Patient eligibility

Patients with an advanced or a recurrent non-small cell lung cancer who failed to respond to the standard therapy were enrolled in these two trials. Eligibility criteria were as follows: (1) patients who had an HLA-A*2402 allele evaluated using DNA genotyping; (2) adequate bone-marrow, cardiac, pulmonary, hepatic and renal functions including a white blood cell count of 1500-15000/mm^3^, a platelet count of >75 000/mm^3^, total bilirubin of < three times that of the institutional normal upper limit, levels of aspartate aminotransferase, alanine aminotransferase, and alkaline phosphatase of < three times that of the institutional normal upper limits, and levels of creatinine of < two times the institutional normal upper limit; (3) no other therapy for lung cancer within 4 weeks prior to the initiation of the trial; (4) an ECOG performance status of 0–2; and (f) an age of ≥20 years. The exclusion criteria for patients participating in the two clinical trials were as follows: (1) pregnancy (including women of childbearing potential); (2) breast feeding; (3) bleeding disorder; (4) infections requiring antibiotics treatment; (5) concomitant treatment with steroid or immunosuppressant; and (6) decision of unsuitableness by principal investigator or physician-in-charge.

### Peptides

The amino acid sequences of the peptides used were RYCNLEGPPI (URLC19-177), VYGIRLEHF (CDCA1-56), SYRNEIAYL (TTK-567), TLFWLLLTL (VEGFR1-770) and RFVPDGNRI (VEGFR2-169); these were expected to bind to an HLA-A24 molecule. These peptides were synthesized as GMP grade as described elsewhere [10-13]. The purity (>97%) and identity of the peptides were determined using analytical high-performance liquid chromatography and mass spectrometry analysis, respectively. Peptides were dissolved in dimethyl-sulfoxide at the concentration of 20 mg/ml and stored at −80°C.

### Enzyme-linked immunospot (ELISPOT) assay

Specific CTL response was measured using an ELISPOT assay following *in vitro* sensitization. Frozen peripheral blood mononuclear cells (PBMCs) isolated from each patient were thawed, and the viability was confirmed to be more than 90%. 500,000 PBMC cells from each patient were cultured with 10 mg/ml of respective peptide and 100 IU/ml of IL-2 (Novartis, Emeryville, CA, USA) at 37°C for two weeks (each peptide was added to the culture medium on days 0 and 7). After CD4^+^ cell depletion using a Dynal CD4-positive isolation kit (Invitrogen, Carlsbad, CA, USA), the IFN-γ ELISPOT assay was performed using a Human IFN-γ ELISpot PLUS kit (MabTech, Nacka Strand, Sweden) according to the manufacturers’ instructions. Briefly, HLA-A*2402-positive B-lymphoblast TISI cells (IHWG Cell and Gene Bank, Seattle, WA, USA) were incubated with 20 mg/ml of each peptide overnight, then the peptide in the media was washed out to prepare the peptide-pulsed TISI cells as stimulator cells. Prepared CD4-negative cells were cultured with the peptide-pulsed TISI cells (2 × 10^4^ cells/well) at the ratio of responder cells and stimulator cells (R/S ratio) of 1:1, 1:2, 1:4 and 1:8 on 96-well plates at 37°C overnight. Non-peptide-pulsed TISI cells were used as negative controls. To confirm the IFN-γ productivity, responder cells (2.5 × 10^3^ cells/well) were stimulated with PMA (66 ng/ml) and ionomycin (3 mg/ml) without stimulator cells overnight, and then applied to the IFN-γ ELISPOT assay. All ELISPOT assays were performed in triplicate wells. The plates were analyzed using the automated ELISPOT reader, ImmunoSPOT S4 (Cellular Technology Ltd, Shaker Heights, OH, USA) and ImmunoSpot Professional Software Version 5.0 (Cellular Technology Ltd). The number of peptide specific spots was calculated by subtracting the spot number in the control well from the spot number in well with peptide-pulsed TISI cells. Antigen specific CTL responses were classified into 4 groups (−, +, ++ or +++) according to a previously reported protocol [14]. If the CTLs were indicated as +, we judged them as being positive in this study. The quality of our ELISPOT assay was ranked at the average level by the ELISPOT panel of Cancer Immunotherapy Consortium (CIC; http://cvc.assaymgmt.webbasix.com).

### Flow cytometrical analysis

The presence of CTLs with peptide-specific T cell receptor was analyzed using a FACS-CantoII (Becton Dickinson, San Jose, CA, USA), using VEGFR1 or VEGFR2-derived epitope peptide-MHC dextramer-PE (Immudex, Copenhagen, Denmark), CDCA1-derived epitope peptide-MHC pentamer-PE (ProImmune Ltd., Oxford, UK), or URLC10-derived epitope peptide-MHC tetramer-PE (Medical & Biological Laboratories Co., Ltd., Nagoya, Japan) according to the manufacturers’ instructions. HIV-derived epitope peptide (RYLRDQQLL)-MHC dextramer, pentamer or tetramer-PE was used as a negative control. Briefly, cells were incubated with the peptide-MHC dextramer, pentamer or tetramer-PE for 10 min at room temperature, and then treated with FITC-conjugated anti-human CD8 mAb, APC-conjugated anti-human CD3 mAb, PE-Cy7-conjugated anti-human CD4 mAb, and 7-AAD (BD Pharmingen, San Diego, CA, USA) at 4°C for 20 min.

### Statistical analysis

Statistical analysis for correlation between clinical response and reaction at the injection site (RAI) was performed Fisher’s exact test. Overall survival rates were analyzed using the Kaplan-Meier method, and survival was measured in days from the first vaccination to death. Statistical significance of the survival period was analyzed using the log-rank test.

## Results

### Clinical characteristics of the enrolled patients

The clinical characteristics of the enrolled patients are summarized in Table 1. Eight advanced-stage patients and seven patients with recurrence after surgery were enrolled in the trials. The mean age of these patients was 56.5 years (±7.5 years). Twelve patients were diagnosed as having adenocarcinoma including two cases with sensitive EGFR mutations (Patients 5 and 12), and there was one patient with squamous cell carcinoma, one patient with pleomorphic carcinoma; the remaining patient was diagnosed as having non-histologically-specified non-small cell lung cancer. The patients had received at least one type of chemotherapy regime prior to enrollment as shown in Table 1.

### Feasibility and adverse reactions

The toxicities observed in the 15 patients are summarized in Tables 2 and 3. There was no severe adverse event considered to be related to the vaccination except for local reactions at the injection sites. Although one patient revealed the elevation of hepatic transaminases equivalent to grade 4 toxicity, we judged that this was not due to the vaccine-related toxicity, but was caused by massive liver metastasis.

**Table 2:** Summary of toxicity in Trial 1 using the TTK containing vaccine

**Vaccine doses**	**0.5 mg (n=3)**		**1.0 mg (n=3)**		**3.0 mg (n=3)**		**Total patients (n=9)**	
	**Grade**		**Grade**		**Grade**		**(%)**	
	**1-2**	**3(4)**	**1-2**	**3(4)**	**1-2**	**3(4)**		
Blood/bone marrow								
Anemia	1	0	1	0	2	0	3	(33%)
Leukopenia	0	0	1	0	0	0	1	(11%)
Constitutional symptoms								
Fatigue	1	0	2	0	1	0	4	(44%)
Gastrointestinal								
Nausea/vomiting	0	0	2	0	1	0	3	(33%)
Anorexia	0	1	2	0	0	0	3	(33%)
Constipation	0	0	1	0	0	0	1	(11%)
Dermatology/skin								
Rash	2	0	2	0	3	0	7	(77%)
Pruritus	0	0	1	0	2	0	3	(33%)
Reaction at the injection site	2	0	2	0	3	0	7	(77%)

**Table 3:** Summary of toxicity in Trial 2 using the CDCA1 containing vaccine

**Vaccine doses**	**1.0 mg (n=3)**		**3.0 mg (n=3)**		**Total patients (n=6)**	
	**Grade**		**Grade**		**(%)**	
	**1-2**	**3(4)**	**1-2**	**3(4)**		
Blood/bone marrow						
Anemia	2	0	2	0	4	(67)
Thrombocytopenia	0	0	1	0	1	(17)
Hepatic						
Elevated AST	0	0	0	(1)	1	(17)
Elevated ALT	0	0	0	(1)	1	(17)
Constitutional symptoms						
Fatigue	0	0	3	0	3	(50)
Fever	1	0	1	0	2	(33)
Gastrointestinal						
Nausea/vomiting	0	0	2	0	2	(33)
Anorexia	0	0	2	0	2	(33)
Constipation	1	0	0	0	1	(17)
Dermatology/skin						
Rash	3	0	3	0	6	(100)
Pruritus	3	0	2	0	5	(83)
Reaction at the injection site	3	0	3	0	6	(100)

### Monitoring of immunological responses and clinical response

PBMCs were obtained from all patients before the vaccine treatment and after every course (one course consists of four vaccinations), and in some patients every month after the vaccine treatment had been completed. Using these PBMCs, we analyzed the levels of peptide-specific CTL responses as shown in Table 4 and Additional file 1: Table S2. Immunological responses were found to be relatively weak in the 0.5 mg/body and 1 mg/body groups relative to the 3 mg/body group in Trial 1. Hence, in Trial 2 we deleted the 0.5 mg/body group and administered 1.0 and 3.0 mg/body. In the 3.0 mg/body group, four of a total of six patients in both of the trials revealed strong CTL responses for at least two kinds of peptides.

**Table 4:** Clinical outcome and immunological response

**Patients**	**Vaccination course**	**RECIST**	**PFS† (DAY)**	**OS§ (DAY)**	**T cell response**					**After treatment**
					**LY6K**	**TTK**	**CDCA1**	**R1**	**R2**	
1	1	PD	15	112	-	++		-	++	None
2	1	PD	29	36	-	+		-	++	None
3	1	PD	43	53	-	+		++	+	None
4	1	PD	33	33	-	-		-	-	None
5	2	PD	53	398	-	-		-	-	EGFR-TKI
6	5	SD	86	834	+	-		-	-	RT
7	1	PD	28	276	-	+		-	++	None
8	4	SD	476	476	+++	+++		+++	+++	None
9	25	SD	400	858	+++	+++		+++	++	None
10	9	SD	200	756	+++		+++	+	+++	EGFR-TKI
11	3	PD	60	265	+++		+++	-	+	None
12	19	SD	490	705*	+++		+++	+++	++	Cx
13	4	PD	53	282	++		++	+	-	None
14	6	SD	83	213	+++		+++	+++	+++	None
15	13	SD	316	571*	+++		+++	+	++	Immune**

*: patients still alive; **: another immunotherapy; †PFS: progression free survival; §OS: overall survival.

When we analyzed CTL induction according to performance status (PS), we only detected a strong CTL response in two out of the seven patients with PS 2, while we observed strong CTL responses in five out of the eight patients with PS 0 or 1. In addition, among the seven patients that showed strong CTL responses, six patients were judged as being in a stable condition using RECIST criteria for at least 2 months. On the other hand, among the eight patients who did not reveal a strong CTL response, seven patients showed rapid progression.

**Figure 1:**
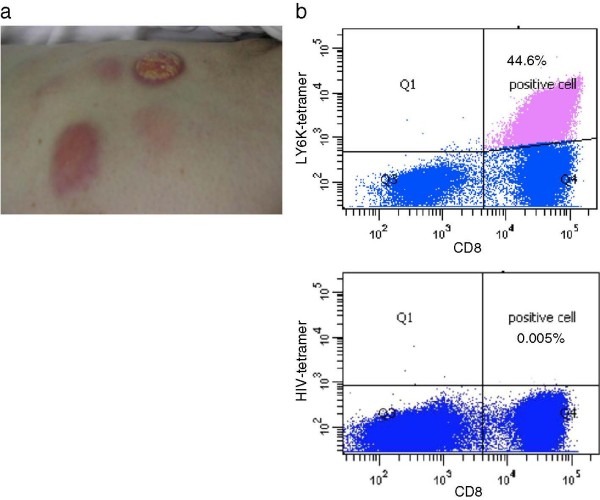
**Strong injection site reaction in patient 8 with positive immune response.** (**a**) Representative picture showing a positive immune reaction at the local injection site (axillary region in patient 8; Grade 2 reaction categorized using CTCAE). (**b**) HLA-tetramer assay showing a very high level of URLC10-specific CD8-positive cells (44.6% of CD8-positive cells) observed after the 4-month vaccine treatment in patient 8.

A representative case of stable disease is shown in Figure 1. Patient 8 had recurrent squamous cell carcinoma with pulmonary metastases. This patient showed relatively strong local reaction at the injection sites and tumors were maintained in a stable condition for 4 months (Figure 1a).

High levels of URLC10-specific CTLs (44.6% of CD8-positive cells) were identified after 4 courses of vaccination.

We also observed the relationship between delayed type hypersensitivity (DTH) as RAI and clinical responses. The stronger the RAI became, the better the clinical responses were, indicating that the RAI seems to be a good biomarker to predict the clinical response (Table 5).

**Table 5:** Reaction at injection site and clinical response

**Clinical response**	**RAI: Grade 0**	**RAI: Grade 1**	**RAI: Grade 2**
Stable disease	0	3	4
Progressive disease	2	6	0

Numbers shown: mean number of patients; RAI: reaction at injection site.

*p*=0.026 (Fisher’s exact test).

### Survival analysis

**Figure 2:**
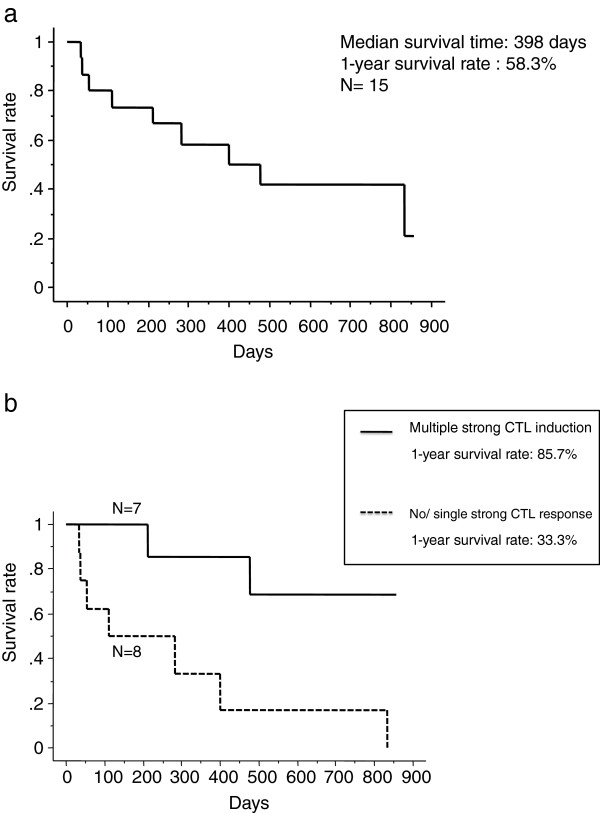
**Survival analysis of patients.** (**a**) Overall survival curve for the fifteen patients analyzed using the Kaplan-Meier method. The median survival time is 398 days and the 1-year survival rate is 58.3%. (**b**) Overall survival curve according to the CTL responses (Kaplan-Meier method). Patients with strong positive CTL responses (+++) to two or more peptides (n=7) had a significantly better prognosis than those revealing a strong CTL response to no or only one peptide (n=8, including several patients who had weak CTL responses with + or ++ against multiple peptides. ) (*p*=0.0176 using the log-rank test). The 1-year survival rates for the group showing a CTL response with multiple peptides and those with no or a single peptide are 85.7% and 33.3%, respectively. As mention above the cutoff levels for CTL were set as (−, +, ++) vs. (+++) in survival analysis.

To clarify the prognostic factors in our vaccine treatment, we further analyzed the survival of patients as shown in Figure 2a, Additional 2: Figure S1 and Table 6. The 1-year survival rate was 58.3% and the median survival period was calculated as being 398 days (56.9 weeks). Sensitive EGFR mutations were found in patients 5 and 12. Patient 10 was treated with Erlotinib as the follow-up therapy, although this patient was found to have no EGFR mutation. The EGFR mutation in patient 5 was found after the vaccine therapy was terminated and was subsequently treated with an EGFR-tyrosine kinase inhibitor (EGFR-TKI). However, because of the poor PS, this patient did not tolerate EGFR-TKI. An EGFR mutation was also detected in patient 12 after the vaccine therapy, but this patient was also treated using cytotoxic chemotherapy because they wished to receive it.

**Table 6:** Clinical and immunological parameters and patient survival

**Parameter**	**1-year survival rate (%)**	**Median survival time (days)**	**P value**
**Total**	**58.3**	**398**	
Age			
>=60y	71.4	476	
< 60y	50	213	0.4159
Sex			
Male	66.7	476	
Female	0	282	0.4797
Performance status			
0-1	100	834	
2	0	112	0.0004
Treatment line			
~2^nd^	72.9	834	
3^rd^~	42.9	112	0.0629
Reaction at injection site			
Strong	75.0	476	
Weak	50.9	398	0.5207
CTL			
Strong	85.7	-	
Weak	33.3	112	0.0176
Regulatory T (%)			
High	57.1	476	
Low	33.3	282	0.3856
C-reactive protein			
>=1.0	25.0	53	
< 1.0	71.6	834	0.0284
Hemoglobin			
Normal	57.1	834	
Low	56.3	398	0.891
Albumin			
Normal	57.1	834	
Low	62.5	398	0.8256
White blood cell count			
High	55.6	-	
Normal	66.7	398	0.7070
Neutrophile (%)			
High	75.0	834	
Low	38.1	282	0.1902
Lymphocyte (%)			
High	50.0	282	
Low	66.7	398	0.5006

As shown in Table 6, PS, CTL response and pre-treatment C-reactive protein (CRP) level (≥1.0 mg/ml) were indicated to be statistically significant prognostic factors (*p*=0.0004, 0.0176 and 0.0284, respectively). Since these three parameters were correlated with each other, further investigation of patients with good PS is essential in the evaluation of the contribution of CTL induction to good prognosis. The number of treatment regimens undergone before enrollment into the vaccine therapy also showed some tendency to influence overall survival (*p*=0.0629). No other laboratory and immunological parameter, including the proportion of regulatory T cells in PBMCs, was significantly correlated with patient survival.

We also analyzed the relationship between patient survival and the number of peptides for which we observed CTL responses. As shown in Figure 2b, patients with CTL induction against multiple peptides had a significantly higher survival rate than those with CTL induction against a single peptide or no peptide, suggesting an advantage in using multiple peptides for cancer treatment.

## Discussion

Among the large number of therapeutic cancer vaccine trials for solid tumors being conducted worldwide, most involve the administration of a single vaccine [15,16]. For lung cancer, two large phase 3 trials using MAGE-A3 or BLP25 are expected to be very promising (ClinicalTrials.gov NCT00480025 and NCT01015443) [5,6]. However, single vaccine therapies in these trials may have some disadvantages as compared with treatment involving a mixture of multiple peptides derived from multiple proteins; one important factor is that antigen expression occurs in a relatively limited proportion of tumors. For example, the expression of MAGE-A3 has been reported in only 40% of cases [17], and in only 24% of Japanese patients [18]. The other important issue is the frequency of CTL induction, the rate of which largely depends on the nature of individual antigens. In fact, two lung cancer studies reported previously shown CTL induction in only 20-53% of the cases treated with vaccines [6,19]. In this regard as recently reported, treatment using multiple vaccine therapy has some advantages owing to the possibility that CTL induction may be higher for one or more antigens [7,8]. Further in renal cell cancer, clinical benefits have been shown lately using a multiple peptide vaccination named IMA901, and a phase 3 study is currently ongoing [20]. In the present study, we have conducted a vaccine trial for lung cancer using multiple peptide vaccines, and observed that the specific CTL responses against one or more epitope peptides were very effective. In only two out of the 15 patients, no CTL induction was observed using any of the four peptides. Although we administered our vaccine treatment to the patients as a second line or later treatment, they achieved a median survival time of 398 days and a 1-year survival rate of 58.3%. Previous major second line trial data regarding NSCLC using a cytotoxic chemotherapeutic drug revealed a median survival time of about ~8 months and a 1-year survival rate of ~30% [21]. Hence, we expect that our vaccine formulation may contribute to an improvement in the prognosis of patients with NSCLC, although further investigation of survival benefit using a larger number of patients is required.

Peptide vaccines used in this trial included peptides that originated from VEGFR1 and VEGFR2 for targeting angiogenesis in tumors. Bevacizmab, an antibody targeting VEGF, has already been used to treat the advanced non-squamous type of NSCLC [22]. Although anti-angiogenic therapy alone does not have sufficient efficacy to induce tumor shrinkage [23], it may support the induction of a strong anti-tumor effect and/or contribute to improved patient survival when it is combined with other therapies [24,25]. Therefore, we considered that the combination of anti-angiogenic peptides with peptides derived from tumor-specific antigen-proteins may cause a synergistic clinical effect in patients with NSCLC. In addition, since HLA molecules are down-regulated in many types of advanced solid cancer including lung cancer [26,27], peptides targeting blood vessels in which HLA molecules are stably expressed should have some anti-tumor effect by reducing the blood supply to tumors.

In our vaccine trial, although we did not observe tumor shrinkage, we observed a possible survival benefit. “Clinical benefit without tumor shrinkage” is considered to be one of the characteristics of cancer vaccine treatment [28]. In fact, the guidance for therapeutic cancer vaccines released from the Food and Drug Administration (FDA) in the United States that was released in 2011 (http://www.fda.gov/downloads/BiologicsBloodVaccines/GuidanceComplianceRegulatoryInformation/Guidances/Vaccines/UCM278673.pdf) mentioned that therapeutic cancer vaccine treatment can provide a survival benefit without evident tumor shrinkage. The FDA guidance further commented that “clinical progression that is asymptomatic and/or is not likely to result in life-threatening complications with further progression (e.g., central nervous system (CNS) metastases or impending fractures from bony metastases) may not be sufficient reason for discontinuation of the administration of a cancer vaccine”. Accumulating evidence has indicated the necessity of establishing novel criteria for the evaluation of clinical response in immunotherapy such as immune-related response criteria (irRC) [28]. Researchers have started using overall survival or relapse-free survival in recently conducted trials as endpoints in immunotherapy clinical trials.

Our data suggested that PS, CTL induction and pre-treatment serum CRP level might be potential predictive markers for vaccine treatment. Extensive and systematic approaches regarding biomarker discovery for vaccine therapy have been carried out [29]. In addition, several prognostic factors possibly related to immunotherapy including clinico-pathological parameters or immunological parameters have been reported [30]. Some previous studies have implicated PS and CTL as good prognostic factors [31,32] in line with our findings. However, although our study has suggested that patients with a higher CRP level (≥1.0 mg/ml) had significantly shorter survival times than those with a lower CRP level, the usefulness of CRP as a prognostic marker has been controversial [33,34].

The US FDA guidance also suggests that cancer vaccine should be administered to patients at an earlier stage, at which the immune system has not been heavily damaged by cytotoxic anti-cancer drugs. In this regard, administration of vaccine therapy should be more appropriate as an adjuvant treatment after surgery, or as an early phase treatment after relapse of the disease in combination with or without chemotherapy.

In summary, we conducted phase I trials with multiple peptide vaccines for patients with NSCLC. These vaccine treatments were well tolerated and prolongation of patient survival owing to vaccine treatment might be expected. We believe that vaccine treatment using multiple peptides is likely to be very promising, although this should be validated by further advanced-phase clinical trials.

## Competing interests

The authors declare that they have no competing interests.

## Authors’ contributions

HS participated as principle investigator of the study. HS, Mitsunori H, YS, TK, KI and MG participated in the design and coordination of the study, data acquisition and analysis and helped draft the manuscript. KT participated as the main coordinator and investigator regarding the immunological data analysis and evaluation. MF, TY, SM, NO, HY, TH, AY, JO and MH participated in the clinical data acquisition and evaluation, and helped draft the manuscript. All authors read and approved the final manuscript.

## Supplementary Material

Additional file 1: Table S2 Summary of Elispot assay data, before, post 1 course and post 2 course vaccination. Click here for file

Additional file 2: Figure S1 (**A**) Overall survival analysis according to patient ECOG performance status. Patients with a good PS (PS: 0, 1) had a significantly higher survival rate than patients with a poor PS (PS: 2) (*p*<0.0001 using the log rank test). (**B**) Overall survival curve according to the CTL responses in the good PS group (PS: 0, 1) (Kaplan-Meier method). Patients with positive CTL responses to two or more peptides (n=5) had a relatively better prognosis than those revealing a CTL response to no or one peptide, although the difference was not significant (n=3; *p*=0.09). Click here for file

## References

[B1] Jemal A Bray F Center MM Ferlay J Ward E Forman D Global cancer statistics CA Cancer J Clin 2011 61 69 90 10.3322/caac.20107 21296855

[B2] Bach PB Mirkin JN Oliver TK Azzoli CG Berry DA Brawley OW Byers T Colditz GA Gould MK Jett JR Sabichi AL Smith-Bindman R Wood DE Qaseem A Detterbeck FC Benefits and harms of CT screening for lung cancer: a systematic review JAMA 2012 307 2418 2429 22610500 10.1001/jama.2012.5521 PMC3709596

[B3] Min JH Lee HY Lim H Ahn MJ Park K Chung MP Lee KS Drug-induced interstitial lung disease in tyrosine kinase inhibitor therapy for non-small cell lung cancer: a review on current insight Cancer Chemother Pharmacol 2011 68 1099 1109 10.1007/s00280-011-1737-2 21913033

[B4] Ricciardi S Tomao S de Marinis F Toxicity of targeted therapy in non-small-cell lung cancer management Clin Lung Cancer 2009 10 28 35 10.3816/CLC.2009.n.004 19289369

[B5] Vansteenkiste J Zielinski M Linder A Dahabre J Esteban E Malinowski W Jassem J Passlick B Lehmann F Brichard VG Final results of a multi-center, double-blind, randomized, placebo-controlled phase II study to assess the efficacy of MAGE-A3 immunotherapeutic as adjuvant therapy in stage IB/II non-small cell lung cancer (NSCLC) [abstract] J Clin Oncol 2007 25 s7554

[B6] Butts C Murray N Maksymiuk A Goss G Marshall E Soulières D Cormier Y Ellis P Price A Sawhney R Davis M Mansi J Smith C Vergidis D Ellis P MacNeil M Palmer M Randomized phase IIB trial of BLP25 liposome vaccine in stage IIIB and IV non-small-cell lung cancer J Clin Oncol 2005 23 6674 6681 10.1200/JCO.2005.13.011 16170175

[B7] Bae J Smith R Daley J Mimura N Tai YT Anderson KC Munshi NC Myeloma-specific multiple peptides able to generate cytotoxic T lymphocytes: A potential therapeutic application in multiple myeloma and other plasma cell disorders Clin Cancer Res 2012 18 4850 4860 10.1158/1078-0432.CCR-11-2776 22753586 PMC3839582

[B8] Kono K Mizukami Y Daigo Y Takano A Masuda K Yoshida K Tsunoda T Kawaguchi Y Nakamura Y Fujii H Vaccination with multiple peptides derived from novel cancer-testis antigens can induce specific T-cell responses and clinical responses in advanced esophageal cancer Cancer Sci 2009 100 1502 1509 10.1111/j.1349-7006.2009.01200.x 19459850 PMC11158753

[B9] Ishikawa N Takano A Yasui W Inai K Nishimura H Ito H Miyagi Y Nakayama H Fujita M Hosokawa M Tsuchiya E Kohno N Nakamura Y Daigo Y Cancer-testis antigen lymphocyte antigen 6 complex locus K is a serologic biomarker and a therapeutic target for lung and esophageal carcinomas Cancer Res 2007 67 11601 11611 10.1158/0008-5472.CAN-07-3243 18089789

[B10] Suda T Tsunoda T Daigo Y Nakamura Y Tahara H Identification of human leukocyte antigen HLA-A24-restricted epitope-peptides derived from gene products up-regulated in lung and esophageal cancers as novel targets for immunotherapy Cancer Sci 2007 98 1803 1808 10.1111/j.1349-7006.2007.00603.x 17784873 PMC11159329

[B11] Ishizaki H Tsunoda T Wada S Yamauchi M Shibuya M Tahara H Inhibition of tumor growth with antiangiogenic cancer vaccine using epitope peptides derived from human vascular endothelial growth factor receptor 1 Clin Cancer Res 2006 12 5841 5849 10.1158/1078-0432.CCR-06-0750 17020992

[B12] Hayama S Daigo Y Kato T Ishikawa N Yamabuki T Miyamoto M Ito T Tsuchiya E Kondo S Nakamura Y Activation of CDCA1-KNTC2, members of centromere protein complex, involved in pulmonary carcinogenesis Cancer Res 2006 66 10339 10348 10.1158/0008-5472.CAN-06-2137 17079454

[B13] Wada S Tsunoda T Baba T Primus FJ Kuwano H Shibuya M Tahara H Rationale for antiangiogenic cancer therapy with vaccination using epitope peptides derived from human vascular endothelial growth factor receptor 2 Cancer Res 2005 65 4939 4946 10.1158/0008-5472.CAN-04-3759 15930316

[B14] Kono K Iinuma H Akutsu Y Tanaka H Hayashi N Uchikado Y Noguchi T Fujii H Okinaka K Fukushima R Matsubara H Ohira M Baba H Natsugoe S Kitano S Takeda K Yoshida K Tsunoda T Nakamura Y Multicenter, phase II clinical trial of cancer vaccination for advanced esophageal cancer with three peptides derived from novel cancer-testis antigens J Transl Med 2012 10 141 149 10.1186/1479-5876-10-141 22776426 PMC3403921

[B15] Shepherd FA Douillard JY Blumenschein GR Jr Immunotherapy for non-small cell lung cancer: novel approaches to improve patient outcome J Thorac Oncol 2011 6 1763 1773 10.1097/JTO.0b013e31822e28fc 21876456

[B16] Kabaker K Shell K Kaufman HL Vaccines for colorectal cancer and renal cell carcinoma Cancer J 2011 17 283 293 10.1097/PPO.0b013e318232ff44 21952277

[B17] Sienel W Varwerk C Linder A Kaiser D Teschner M Delire M Stamatis G Passlick B Melanoma associated antigen (MAGE)-A3 expression in Stages I and II non-small cell lung cancer: results of a multi-center study Eur J Cardiothorac Surg 2004 25 131 134 10.1016/j.ejcts.2003.09.015 14690745

[B18] Shigematsu Y Hanagiri T Shiota H Kuroda K Baba T Mizukami M So T Ichiki Y Yasuda M So T Takenoyama M Yasumoto K Clinical significance of cancer/testis antigens expression in patients with non-small cell lung cancer Lung Cancer 2010 68 105 110 10.1016/j.lungcan.2009.05.010 19545928

[B19] Nemunaitis J Dillman RO Schwarzenberger PO Senzer N Cunningham C Cutler J Tong A Kumar P Pappen B Hamilton C DeVol E Maples PB Liu L Chamberlin T Shawler DL Fakhrai H Phase II study of belagenpumatucel-L, a transforming growth factor beta-2 antisense gene-modified allogeneic tumor cell vaccine in non-small-cell lung cancer J Clin Oncol 2006 24 4721 4730 10.1200/JCO.2005.05.5335 16966690

[B20] Walter S Weinschenk T Stenzl A Zdrojowy R Pluzanska A Szczylik C Staehler M Brugger W Dietrich PY Mendrzyk R Hilf N Schoor O Fritsche J Mahr A Maurer D Vass V Trautwein C Lewandrowski P Flohr C Pohla H Stanczak JJ Bronte V Mandruzzato S Biedermann T Pawelec G Derhovanessian E Yamagishi H Miki T Hongo F Takaha N Multipeptide immune response to cancer vaccine IMA901 after single-dose cyclophosphamide associates with longer patient survival Nat Med 2012 18 1254 1261 10.1038/nm.2883 22842478

[B21] Hanna N Shepherd FA Fossella FV Pereira JR De Marinis F von Pawel J Gatzemeier U Tsao TC Pless M Muller T Lim HL Desch C Szondy K Gervais R Shaharyar M Manegold C Paul S Paoletti P Einhorn L Bunn PA Jr Randomized phase III trial of pemetrexed versus docetaxel in patients with non-small-cell lung cancer previously treated with chemotherapy J Clin Oncol 2004 22 1589 1597 10.1200/JCO.2004.08.163 15117980

[B22] Ulahannan SV Brahmer JR Antiangiogenic agents in combination with chemotherapy in patients with advanced non-small cell lung cancer Cancer Invest 2011 29 325 337 10.3109/07357907.2011.554476 21469981 PMC3082199

[B23] Yang JC Haworth L Sherry RM Hwu P Schwartzentruber DJ Topalian SL Steinberg SM Chen HX Rosenberg SA A randomized trial of bevacizumab, an anti-vascular endothelial growth factor antibody, for metastatic renal cancer N Engl JMed 2003 349 427 434 10.1056/NEJMoa021491 12890841 PMC2275324

[B24] Sandler A Gray R Perry MC Brahmer J Schiller JH Dowlati A Lilenbaum R Johnson DH Paclitaxel-carboplatin alone or with bevacizumab for non-small-cell lung cancer N Engl J Med 2006 355 2542 2550 10.1056/NEJMoa061884 17167137

[B25] Reck M von Pawel J Zatloukal P Ramlau R Gorbounova V Hirsh V Leighl N Mezger J Archer V Moore N Manegold C Phase III trial of cisplatin plus gemcitabine with either placebo or bevacizumab as first-line therapy for nonsquamous non-small-cell lung cancer J Clin Oncol 2009 27 1227 1234 10.1200/JCO.2007.14.5466 19188680

[B26] Bubeník J MHC class I down-regulation: tumour escape from immune surveillance? (review) Int J Oncol 2004 25 487 491 15254748

[B27] Kikuchi E Yamazaki K Torigoe T Cho Y Miyamoto M Oizumi S Hommura F Dosaka-Akita H Nishimura M HLA class I antigen expression is associated with a favorable prognosis in early stage non-small cell lung cancer Cancer Sci 2007 98 1424 1430 10.1111/j.1349-7006.2007.00558.x 17645781 PMC11159758

[B28] Wolchok JD Hoos A O’Day S Weber JS Hamid O Lebbé C Maio M Binder M Bohnsack O Nichol G Humphrey R Hodi FS Guidelines for the evaluation of immune therapy activity in solid tumors: immune-related response criteria Clin Cancer Res 2009 15 7412 7420 10.1158/1078-0432.CCR-09-1624 19934295

[B29] Butterfield LH Disis ML Fox BA Lee PP Khleif SN Thurin M Trinchieri G Wang E Wigginton J Chaussabel D Coukos G Dhodapkar M Håkansson L Janetzki S Kleen TO Kirkwood JM Maccalli C Maecker H Maio M Malyguine A Masucci G Palucka AK Potter DM Ribas A Rivoltini L Schendel D Seliger B Selvan S Slingluff CL Jr Stroncek DF A systematic approach to biomarker discovery; preamble to “the iSBTc-FDA taskforce on immunotherapy biomarkers” J Transl Med 2008 6 81 91 10.1186/1479-5876-6-81 19105846 PMC2630944

[B30] Quoix E Ramlau R Westeel V Papai Z Madroszyk A Riviere A Koralewski P Breton JL Stoelben E Braun D Debieuvre D Lena H Buyse M Chenard MP Acres B Lacoste G Bastien B Tavernaro A Bizouarne N Bonnefoy JY Limacher JM Therapeutic vaccination with TG4010 and first-line chemotherapy in advanced non-small-cell lung cancer: a controlled phase 2B trial Lancet Oncol 2011 12 1125 1133 10.1016/S1470-2045(11)70259-5 22019520

[B31] Sawada Y Yoshikawa T Nobuoka D Shirakawa H Kuronuma T Motomura Y Mizuno S Ishii H Nakachi K Konishi M Nakagohri T Takahashi S Gotohda N Takayama T Yamao K Uesaka K Furuse J Kinoshita T Nakatsura T Phase I trial of a glypican-3-derived peptide vaccine for advanced hepatocellular carcinoma: Immunologic evidence and potential for improving overall survival Clin Cancer Res 2012 18 3686 3696 10.1158/1078-0432.CCR-11-3044 22577059

[B32] Dillman RO Fogel GB Cornforth AN Selvan SR Schiltz PM DePriest C Features associated with survival in metastatic melanoma patients treated with patient-specific dendritic cell vaccines Cancer Biother Radiopharm 2011 26 407 415 10.1089/cbr.2011.0973 21812653

[B33] Lukaszewicz-Zając M Mroczko B Gryko M Kędra B Szmitkowski M Comparison between clinical significance of serum proinflammatory proteins (IL-6 and CRP) and classic tumor markers (CEA and CA 19–9) in gastric cancer Clin Exp Med 2011 11 89 96 10.1007/s10238-010-0114-5 20938721 PMC3087107

[B34] Kwon KA Kim SH Oh SY Lee S Han JY Kim KH Goh RY Choi HJ Park KJ Roh MS Kim HJ Kwon HC Lee JH Clinical significance of preoperative serum vascular endothelial growth factor, interleukin-6, and C-reactive protein level in colorectal cancer BMC Cancer 2010 10 203 210 10.1186/1471-2407-10-203 20465852 PMC2886042

