# Supplementary material for: Multiple therapeutic peptide vaccines consisting of combined novel cancer testis antigens and anti-angiogenic peptides for patients with non-small cell lung cancer
Source: J Transl Med. 2013 Apr 11;11:97. doi: 10.1186/1479-5876-11-97 (PMC3639131; doi:10.1186/1479-5876-11-97)
Supplement: Additional file 1: Table S2 — Summary of Elispot assay data, before, post 1 course and post 2 course vaccination. [file 1479-5876-11-97-S1.docx]

| Patients |  | LY6K | TTK | CDCA1 | R1 | R2 |
| --- | --- | --- | --- | --- | --- | --- |
| 1 | Pre* | − | − |  | − | ＋＋ |
|  | Post 1** | − | ＋＋ |  | − | ＋＋ |
|  | Post 2*** | NA§ | NA |  | NA | NA |
| 2 | Pre | − | ＋ |  | − | ＋ |
|  | Post 1 | − | ＋ |  | − | ＋＋ |
|  | Post 2 | NA | NA |  | NA | NA |
| 3 | Pre | − | ＋＋ |  | − | ＋＋ |
|  | Post 1 | − | ＋ |  | ＋＋ | ＋ |
|  | Post 2 | NA | NA |  | NA | NA |
| 4 | Pre | − | − |  | − | − |
|  | Post 1 | − | − |  | − | − |
|  | Post 2 | NA | NA |  | NA | NA |
| 5 | Pre | − | − |  | − | − |
|  | Post 1 | − | − |  | − | − |
|  | Post 2 | − | − |  | − | − |
| 6 | Pre | − | − |  | − | − |
|  | Post 1 | − | − |  | − | − |
|  | Post 2 | ＋ | − |  | − | − |
| 7 | Pre | ＋ | − |  | − | − |
|  | Post 1 | − | ＋ |  | − | ＋＋ |
|  | Post 2 | NA | NA |  | NA | NA |

Supplementary Table

Summary of Elispot assay data, before, post 1 course and post 2 course vaccination.

Continued

| 8 | Pre | ＋ | − |  | − | ＋ |
| --- | --- | --- | --- | --- | --- | --- |
|  | Post 1 | ＋＋ | ＋＋ |  | − | ＋＋ |
|  | Post 2 | ＋＋＋ | ＋ |  | ＋＋＋ | ＋ |
| 9 | Pre | − | ＋ |  | ＋ | − |
|  | Post 1 | − | − |  | − | ＋ |
|  | Post 2 | − | − |  | ＋＋ | ＋ |
| 10 | Pre | − |  | − | − | − |
|  | Post 1 | ＋＋ |  | − | − | − |
|  | Post 2 | ＋＋＋ |  | − | − | − |
| 11 | Pre | − |  | ＋＋ | ＋ | − |
|  | Post 1 | ＋＋＋ |  | − | − | − |
|  | Post 2 | ＋＋＋ |  | ＋＋＋ | − | − |
| 12 | Pre | − |  | − | − | − |
|  | Post 1 | ＋＋＋ |  | − | − | − |
|  | Post 2 | ＋＋＋ |  | ＋＋＋ | ＋＋ | ＋ |
| 13 | Pre | − |  | − | − | − |
|  | Post 1 | − |  | − | − | − |
|  | Post 2 | − |  | − | − | − |
| 14 | Pre | − |  | − | − | ＋ |
|  | Post 1 | − |  | ＋ | ＋＋＋ | − |
|  | Post 2 | − |  | ＋＋＋ | − | ＋＋ |
| 15 | Pre | − |  | − | ＋ | − |
|  | Post 1 | − |  | − | − | − |
|  | Post 2 | ＋ |  | ＋ | − | ＋ |

*Pre: Data of pre-vaccination. **Post 1: Data of post 1 course of vaccination. ***Post 1: Data of post 2 course of vaccination.

§NA: Not available

†
