# Supplementary material for: Multiple therapeutic peptide vaccines consisting of combined novel cancer testis antigens and anti-angiogenic peptides for patients with non-small cell lung cancer
Source: J Transl Med. 2013 Apr 11;11:97. doi: 10.1186/1479-5876-11-97 (PMC3639131; doi:10.1186/1479-5876-11-97)
Supplement: Additional file 2: Figure S1 — (A) Overall survival analysis according to patient ECOG performance status. Patients with a good PS (PS: 0, 1) had a significantly higher survival rate than patients with a poor PS (PS: 2) (p<0.0001 using the log rank test). (B) Overall survival curve according to the CTL responses in the good PS group (PS: 0, 1) (Kaplan-Meier method). Patients with positive CTL responses to two or more peptides (n=5) had a relatively better prognosis than those revealing a CTL response to no or one peptide, although the difference was not significant (n=3; p=0.09). [file 1479-5876-11-97-S2.pptx]

## Slide 1
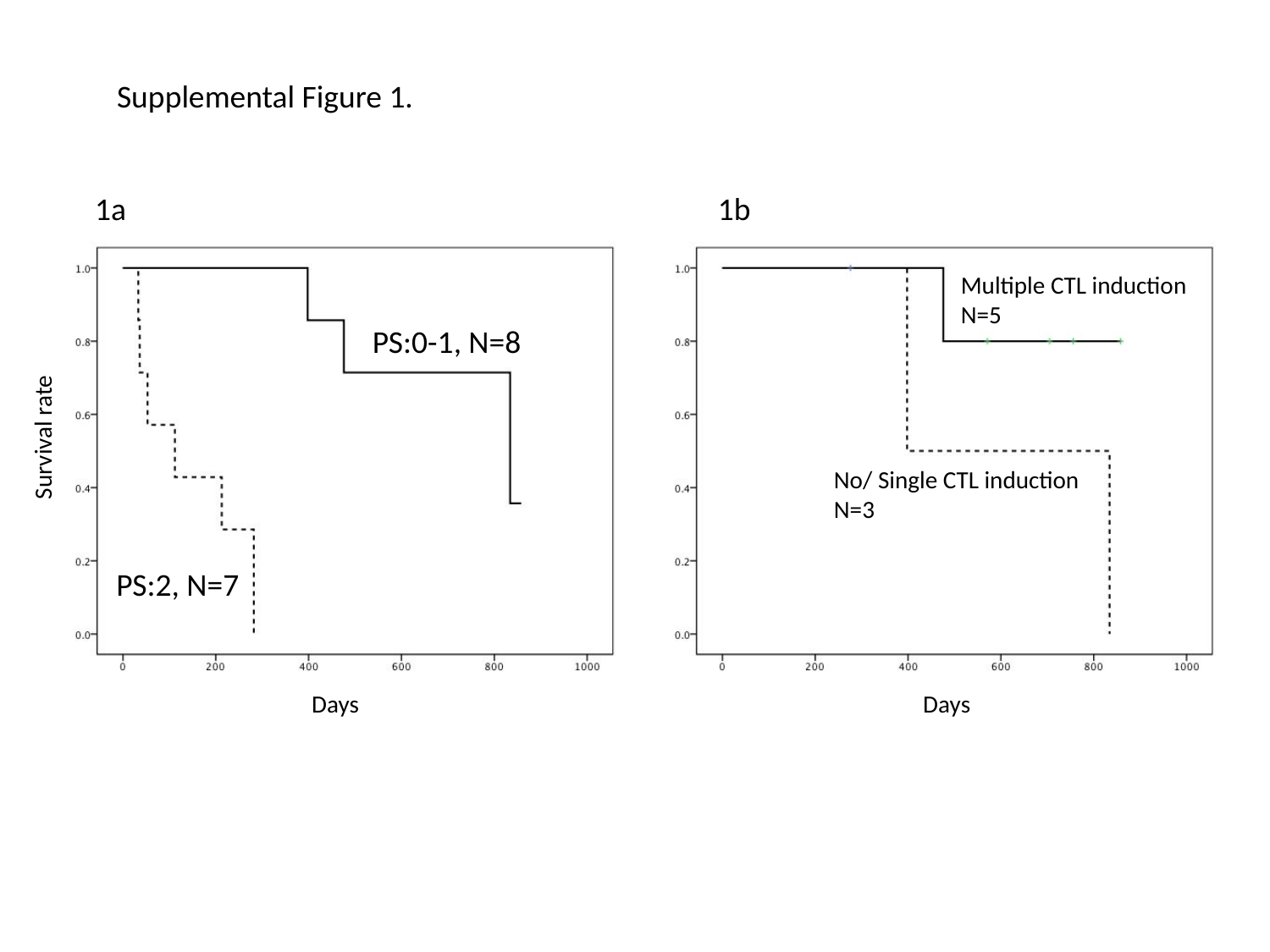

Supplemental Figure 1.
1a
1b
Multiple CTL induction
N=5
PS:0-1, N=8
Survival rate
No/ Single CTL induction
N=3
PS:2, N=7
Days
Days
